# Profiles of Extracellular miRNA in Cerebrospinal Fluid and Serum from Patients with Alzheimer's and Parkinson's Diseases Correlate with Disease Status and Features of Pathology

**DOI:** 10.1371/journal.pone.0094839

**Published:** 2014-05-05

**Authors:** Kasandra Burgos, Ivana Malenica, Raghu Metpally, Amanda Courtright, Benjamin Rakela, Thomas Beach, Holly Shill, Charles Adler, Marwan Sabbagh, Stephen Villa, Waibhav Tembe, David Craig, Kendall Van Keuren-Jensen

**Affiliations:** 1 Neurogenomics, Translational Genomics Research Institute (TGen), Phoenix, Arizona, United States of America; 2 Neurology, Banner Sun Health Research Institute, Sun City, Arizona, United States of America; 3 Neurology, Mayo Clinic, Scottsdale, Arizona, United States of America; University of Melbourne, Australia

## Abstract

The discovery and reliable detection of markers for neurodegenerative diseases have been complicated by the inaccessibility of the diseased tissue- such as the inability to biopsy or test tissue from the central nervous system directly. RNAs originating from hard to access tissues, such as neurons within the brain and spinal cord, have the potential to get to the periphery where they can be detected non-invasively. The formation and extracellular release of microvesicles and RNA binding proteins have been found to carry RNA from cells of the central nervous system to the periphery and protect the RNA from degradation. Extracellular miRNAs detectable in peripheral circulation can provide information about cellular changes associated with human health and disease. In order to associate miRNA signals present in cell-free peripheral biofluids with neurodegenerative disease status of patients with Alzheimer's and Parkinson's diseases, we assessed the miRNA content in cerebrospinal fluid and serum from postmortem subjects with full neuropathology evaluations. We profiled the miRNA content from 69 patients with Alzheimer's disease, 67 with Parkinson's disease and 78 neurologically normal controls using next generation small RNA sequencing (NGS). We report the average abundance of each detected miRNA in cerebrospinal fluid and in serum and describe 13 novel miRNAs that were identified. We correlated changes in miRNA expression with aspects of disease severity such as Braak stage, dementia status, plaque and tangle densities, and the presence and severity of Lewy body pathology. Many of the differentially expressed miRNAs detected in peripheral cell-free cerebrospinal fluid and serum were previously reported in the literature to be deregulated in brain tissue from patients with neurodegenerative disease. These data indicate that extracellular miRNAs detectable in the cerebrospinal fluid and serum are reflective of cell-based changes in pathology and can be used to assess disease progression and therapeutic efficacy.

## Introduction

The ability to meaningfully profile peripheral biofluids to monitor and gain insights about the underlying severity of central nervous system pathology would bring significant benefits to monitoring disease progression and treatment efficacy. Development of diagnostic tests and preventative and treatment therapies for neurodegenerative diseases is encumbered by the complexity of pathomechanisms underlying neurodegenerative diseases, as well as the difficulty of achieving an accurate diagnosis in early, asymptomatic stages of disease. Whereas several genes have been linked to rare monogenic forms of Alzheimer's disease (AD) and Parkinson's disease (PD), molecular mechanisms underlying sporadic forms of the disease are complex and largely unknown [1], [2].

AD is an age-related, chronic, neurodegenerative disorder characterized by gradual dementia and deteriorated higher cognitive functions including language and behavior [3]. Similarly to AD, PD is a progressive neurodegenerative disorder affecting approximately 1–2% of individuals over 60 years of age [4]. Cardinal clinical features of PD are rigidity, resting tremor, bradykinesia and postural instability [3]. As PD advances, up to 80% of patients develop dementia.

Histopathologically, the AD brain is characterized by deposition of both neuritic plaques composed of amyloid- 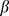 (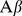) peptide and hyperphosphorylated forms of the microtubule-associated protein Tau that create neurofibrillary tangles (NFTs) [2]. Neurons of PD subjects exhibit abnormal accumulation of cytoplasmic inclusions consisting mainly of 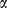 -synuclein, a protein whose aggregation forms insoluble fibrils, Lewy Bodies [3]. To complicate the detection of AD and PD, age-matched cognitively normal individuals have low levels of plaque and tangle formation, as do most PD patients.

An important emerging level of pathophysiological complexity underlying neurodegenerative disorders is derived from miRNA gene regulation [5], [6]. MiRNAs represent a class of endogenous, stable, non-coding RNA molecules involved in post-transcriptional regulation of target gene expression. Biogenesis of mature miRNA occurs through a multi-step process that starts in the nucleus with endonucleolytic cleavage of the primary miRNA transcript, and ends with a ∼20–25 nucleotides long single stranded mature miRNA (miRNA) in the cytosol. The binding of miRNA with imperfect complementarity to target mRNAs leads to a reduced protein expression by either degradation of the RNA or translational arrest [7]. Discovery of miRNA regulatory potential has significantly broadened our knowledge of preferential gene expression in the central nervous system. Half of the identified tissue specific miRNAs are brain or brain-region specific, promoting homeostatic functions on brain gene expression [8], [9]. Several age-related disease studies suggest differential expression of several miRNAs in the human brain, some of which regulate the expression of genes known to be associated with neurodegeneration [10], [11], [12]. More importantly, abnormal expression of miRNAs have been detected in cellular dysfunction and disease, including AD and PD [1], [6], [13], [14], [15].

The concept that peripheral biofluids, such as cerebrospinal fluid (CSF) and blood serum (SER), contain markers of central nervous system disorders has become an active area of research. Circulating cell-free RNAs, as indicators (snapshots) of disease-relevant information, are carried to the periphery and are attractive candidates for monitoring central nervous system disease. The miRNA changes associated with neurodegenerative disease that are detectable in the periphery have not been appreciably profiled and compared in the CSF and SER of AD and PD patients. Profiling cell-free miRNA may reduce interfering miRNA signals from blood cells and immune cells [16]. In addition, there has not been an extensive study to correlate peripheral miRNAs with corresponding postmortem neuropathology characterization.

Recent advances in library sample preparation and analytical methods have introduced new protocols that allow miRNA profiling by next generation sequencing (NGS) from CSF and SER [17]. In this study, we used NGS to investigate the expression patterns of the known miRNAs listed in miRBase (V18) in acellular fluids from postmortem subjects with verification of Alzheimer's or Parkinson's disease neuropathology, and neurologically normal control subjects. We compared the detectible miRNAs in CSF and SER. Postmortem autopsy data on brain tissue revealed the severity and extent of neuropathology, which we were able to correlate with miRNA status in biofluids. As a potential biofluid of choice for CNS disease, human CSF has the advantage to reflect a more stable signature of the brain due to its proximity to the diseased tissue. However, unless there is a significant precedent to submit a patient to a lumbar puncture, most patients are reluctant. Serum is less invasive and more readily available, but also contains miRNA signals from all tissues in the body. One goal of this study was to ascertain the advantages of CSF compared with SER for the detection of Alzheimer's and Parkinson's disease-relevant miRNA. From postmortem patients we were able to profile both the CSF and serum. To determine which fluid has a higher signal-to-noise ratio, we sequenced and analyzed miRNA abundance in paired CSF and SER samples from a cohort consisting of control, AD, and PD subjects. The sample set was used to correlate miRNAs associated with AD and PD pathology that are detectable in peripheral biofluids. We identified AD and PD miRNA signatures, as well as subsets of misregulated miRNAs in connection with regional (Braak stage) and time-dependent characteristics (tangle and plaque load) of AD and PD pathology. Importantly, identical analysis of CSF and SER datasets revealed non-overlapping results, with a potentially more stable miRNA signature derived from the CSF.

One of the advantages to using sequencing to profile the miRNA content is the ability to assess all detectable miRNA expression at once. We used miRDeep2 software [18] to predict novel miRNAs in CSF and SER. We report the differential expression of these putative miRNAs in both CSF and SER across diseases. In addition, we compare our findings with those previously reported for deregulated miRNAs identified in tissue.

This is the first paper to use sequencing to compare the miRNA profile in both CSF and SER from the same individuals. In addition, we sequenced one of the largest miRNA datasets to date, comparing two neurodegenerative diseases. The profiling and sequencing data from this paper are publicly available and represent a significant resource for future evaluations of control, AD and PD biofluids. These data can provide us with information regarding the types of miRNAs detectable in cell-free peripheral biofluids.

## Results

### *miRNA expression profiling*

**Figure 1:**
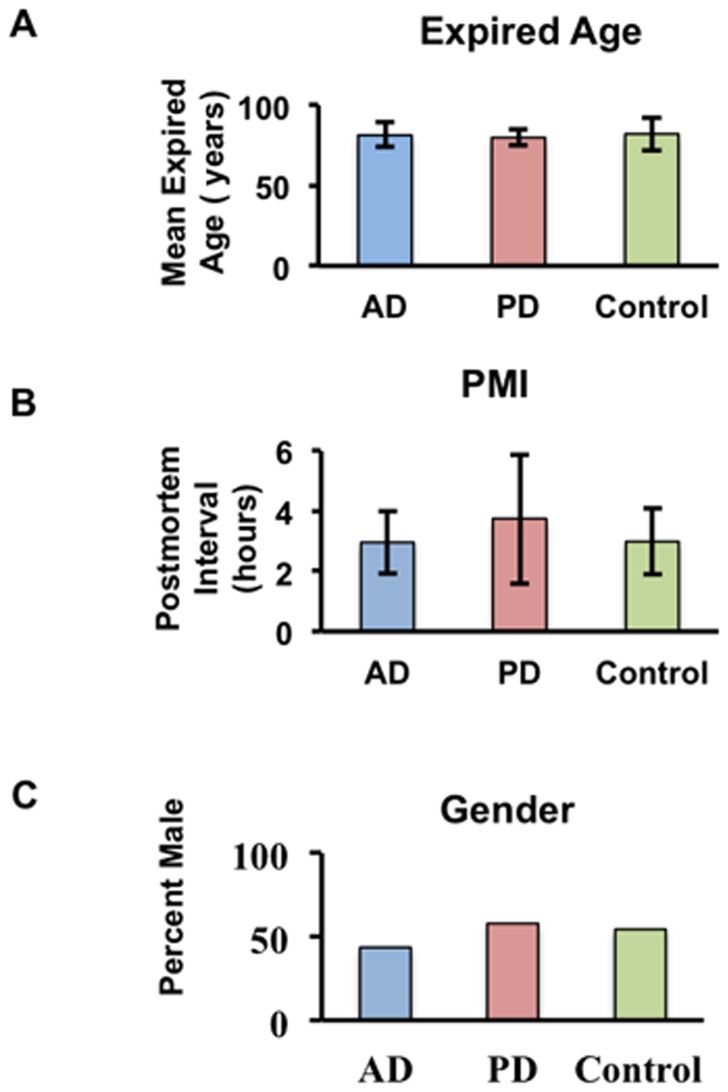
Potential sources of variation for the sample cohort. Three-way ANOVA analysis of variation demonstrates that (**A**) expiration age, (**B**) postmortem interval (PMI) and (**C**) gender do not contribute significant variation to the miRNA expression data.

The principal demographic, postmortem interval, clinical and pathological characteristics of the 69 AD patients, 67 PD patients and 78 control subject samples included in this miRNA profiling study are summarized in **Table S1**. Samples were obtained from the Banner Sun Health Research Institute after thorough evaluation of neuropathology and consisted of AD, PD, and neurologically normal control subjects. Average expired age was comparable across the three groups: controls (82.1 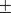 10 years), AD (81.3 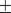 7.7 years) and PD (80.0 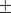 5.1 years) (**Figure 1**). Average disease duration was 7.5 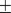 4.1 years for AD patients, and 12.6 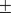 7.9 years for PD subjects. Mean postmortem interval for all samples was approximately 3.1 hours. In most cases, we were able to analyze one CSF and one SER sample from each subject, hence allowing for direct comparison of miRNA signatures for the two biofluids and thereby reducing sample variability. Supporting the consistency of our results, analysis of variance revealed no significant source of variation in the expression data due to age, gender, or postmortem interval (PMI; **Figure S1**).

We conducted miRNA expression profiling of SER and CSF samples using NGS. Small RNA sample preparation for NGS platforms typically require at least 1 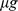 of total RNA as a starting input. This is problematic for SER and CSF samples which contain low levels of total RNA. We modified a protocol for small RNA deep sequencing for samples with low RNA content and small starting volumes, allowing for miRNA NGS expression profiling from CSF and SER [17]. We concentrated our down-stream analysis on the 2228 known miRNAs in miRBase (Version 18). When examining the data from all of our CSF samples simultaneously, we detected 1773 different miRNAs expressed at least once in the CSF samples and 1757 in the SER samples. For our analysis, we reduced these numbers to 428 miRNAs in CSF and 414 miRNAs in SER that had a minimum average of >5 read counts. From the 2228 possible mature miRNAs listed, we removed those that had the same expression patterns across all samples. For example, if hsa-let-7a-5p_hsa-let-7a-1 and hsa-let-7a-5p_hsa-let-7a-2 were present with the same expression profile, hsa-let-7a-5p_hsa-let-7a-2 was considered redundant and removed from further analysis.

Because this is the first paper to sequence and compare the miRNA profile of CSF and SER from the same patients, we provided a list of the 2228 miRNAs used in our analysis and the normalized average number of counts per million detected in each biofluid, from all samples (Table S2).

### *miRNA signature derived from CSF is slightly more stable*

In an effort to determine which biofluid, CSF or SER, has a more stable and consistent miRNA signature associated with disease, we compared the matched CSF and SER data sets derived from AD, PD and control samples. Using consensus clustering analysis and silhouette scores (Figures S1 and S2), the serum data reflected a slightly reduced stability in cluster membership compared to the CSF due to the predominantly unimodal nature of its consensus matrix histogram (Figure S2). However, consensus clustering analysis revealed that there was only a slight improvement in CSF cluster stability in our data sets. Therefore, we report our results for both CSF and SER due to the lack of significant advantage of using either biofluid.

### *miRNAs are differentially expressed in CSF and SER of AD patients*

The samples from AD and age-matched non-affected subjects were subsequently analyzed for differential miRNA content. Based on the distribution of total number of mapped reads (sequence reads that align to known mature miRNAs), we set the threshold for removing samples to those with less than 100,000 mapped reads for CSF and less than 60,000 for SER data. Subsequently, we removed *m* outliers from the following groups: CSF AD (m = 5), CSF Control (m = 5), SER AD (m = 11) and SER Control (m = 10). The remaining samples each had an average of 2,631,443 reads that mapped to known miRNAs for CSF samples and 1,953,105 mapped read counts for SER samples. These samples represent some of the largest depth of coverage in any study to date.

A total of 41 miRNAs were determined to have different expression levels in AD CSF (n = 62) compared with Control CSF (n = 65), corrected for multiple tests with the Benjamini-Hochberg method and normalized mean >5 mapped reads for each group (**Table 1**). There have been many studies identifying deregulated miRNAs in brain tissue from patients with AD compared to neurologically normal controls. Of the 41 significant miRNAs that were expressed differently between the CSF of AD and control subjects, 30 (∼73%) have been previously identified as deregulated in AD: 101-5p, 124-3p, 127-3p, 127-5p, 132-3p, 129-5p, 136-3p, 136-5p, 138-5p, 139-5p, 181a-5p, 181a-3p, 181b-5p, 184, 218-5p, 323a-3p, 326, 329, 377-5p, 381, 410, 433, 488-3p, 495, 708-5p, 769-5p, 874, 9-3p, 9-5p, 95 [6], [19], [20], [21], [22], [23], [24], [25].

**Table 1:** Differentially expressed miRNAs detected in the cerebrospinal fluid (CSF).

Control vs. AD CSF			Control vs. PD CSF			AD vs. PD CSF		
hsa-mirName	FC (log2)	Adjusted p-value	hsa-mirName	FC (log2)	Adjusted p-value	hsa-mirName	FC (log2)	Adjusted p-value
								
miR-124-3p	−1.56	7.15E-07	miR-132-5p^3^	−1.02	3.93E-04	miR-19b-3p	0.98	2.34E-04
miR-138-5p	−1.46	8.54E-07	miR-19a-3p	0.98	3.93E-04	miR-19a-3p	0.94	6.84E-04
miR-127-3p^1,2^	−1.17	1.92E-05	miR-19b-3p	0.92	3.93E-04	miR-127-5p	0.83	1.57E-02
miR-132-3p	−0.89	1.92E-05	miR-485-5p	−1.08	3.93E-04	miR-873-5p	0.82	1.57E-02
miR-127-5p	−1.15	3.09E-05	miR-127-3p^1^	−0.98	4.48E-04	miR-32-5p	0.71	3.06E-02
miR-136-3p^1^	−1.02	3.96E-05	miR-128	−0.85	3.45E-03			
miR-381	−1.14	4.94E-05	miR-409-3p	−0.77	3.45E-03			
miR-101-5p^3^	−0.92	6.72E-05	miR-433^1^	−0.86	6.29E-03			
miR-199b-5p^3^	−1.22	6.72E-05	let-7g-3p^3^	0.82	8.41E-03			
miR-136-5p	−0.91	1.91E-04	miR-370	−0.83	8.41E-03			
miR-184^2^	−0.97	2.69E-04	miR-431-3p^1,3^	−0.81	8.41E-03			
miR-181a-5p	−0.71	3.57E-04	miR-873-3p	−0.83	8.68E-03			
miR-598	−0.96	4.15E-04	miR-136-3p^1^	−0.72	1.20E-02			
miR-218-5p	−0.79	4.93E-04	miR-212-3p	−0.73	2.29E-02			
miR-9-3p	−0.84	4.95E-04	miR-10a-5p^1^	−0.76	2.37E-02			
miR-769-5p	−0.84	6.20E-04	miR-1224-5p	−0.76	3.29E-02			
miR-95^3^	−0.95	6.20E-04	miR-4448	−0.7	4.78E-02			
miR-760	−0.88	8.46E-04						
miR-181a-3p	−0.74	1.01E-03						
miR-181b-5p	−0.75	1.01E-03						
miR-488-3p^3^	−0.88	1.01E-03						
miR-495^3^	−1.07	1.01E-03						
miR-708-3p	−0.84	1.01E-03						
miR-874	−0.75	1.01E-03						
miR-873-5p	−0.81	1.48E-03						
miR-129-5p	−0.84	1.65E-03						
miR-181d	−0.72	1.89E-03						
miR-139-5p	−0.84	1.96E-03						
miR-3200-3p	−0.75	2.77E-03						
miR-431-3p^1,3^	−0.91	3.72E-03						
miR-9-5p	−0.75	4.97E-03						
miR-326	−0.76	5.18E-03						
miR-377-5p^3^	−0.81	6.87E-03						
miR-433^1^	−0.85	7.77E-03						
miR-323a-3p	−0.73	8.53E-03						
miR-134	−0.7	8.98E-03						
miR-329^3^	−0.83	8.98E-03						
miR-10a-5p^1^	−0.84	1.16E-02						
miR-33b-5p	−0.74	1.24E-02						
miR-410	−0.71	1.47E-02						
miR-708-5p	−0.78	1.51E-02						

Sample size for cerebrospinal fluid consisted of 62 AD, 57 PD and 65 control subjects. Results were filtered at adjusted p-value <0.05. The logarithmic base 2 fold change (FC) is relative to the first listed group for each comparison. Significant miRNAs were reported if their normalized base average is greater than 5 mapped reads and 0.7< FC(log2) or FC(log2) <−0.7. Superscript 1 indicates miRNAs that are differentially expressed in both patients with Alzheimer's disease and Parkinson's disease compared to control subjects. Superscript 2 indicates differentially expressed miRNAs in both CSF and SER biofluids for the corresponding analysis. Significant miRNAs with a superscript 3 are in low abundance, with normalized mean <10 mapped reads.

Sample size for serum consisted of 53 AD, n = 50 PD and 62 control subjects. Results were filtered at corrected p-value <0.05 (**Table 2**). We describe only significant differentially expressed miRNAs with an average number of mapped reads greater than 5 and 0.7< FC(log2) or FC(log2) <−0.7. Logarithmic base 2 fold change (FC) is relative to the first listed group for each comparison. Of the 20 differentially expressed miRNAs, we found that 11 (∼55%) were previously reported in the literature: 125a-3p, 125b, 127-3p, 1285, 135a/b, 30c, 21-5p, 219-2-3p, 34c, 375, 873 [2], [6], [25], [26], [27], [28], [29], [30], [31]. The overlap of CSF and SER expressed miRNAs for AD compared to neurologically normal control subject analysis consists of two miRNAs, miR-184 and miR-127-3p. The direction of miR-184 and miR-127-3p expression did not correlate between CSF and SER data. It is interesting to note that the miRNAs expressed differently in the CSF were all significantly down-regulated, whereas 85% of the miRNAs identified in SER were up-regulated compared to neurologically normal age-similar controls.

**Table 2:** Differentially expressed miRNAs detected in the serum (SER).

Control vs. AD SER			Control vs. PD SER			AD vs. PD SER		
hsa-mirName	FC (log2)	Adjusted p-value	hsa-mirName	FC (log2)	Adjusted p-value	hsa-mirName	FC (log2)	Adjusted p-value
								
miR-34b-3p	2.36	1.85E-05	miR-338-3p	1.35	1.06E-03	miR-320a	−1.81	4.64E-05
miR-219-2-3p	1.93	5.37E-04	miR-16-2-3p	−1.09	3.96E-03	miR-320b	−1.84	4.64E-05
miR-22-5p	1.38	5.37E-04	miR-1294	−1.04	1.17E-02	miR-378d	−1.69	4.64E-05
miR-125b-1-3p	1.32	8.03E-04	miR-30e-3p	0.97	1.33E-02	miR-378f	−1.7	4.64E-05
miR-1307-5p	1.39	1.28E-03	miR-30a-3p	0.95	1.54E-02	miR-378a	−1.54	7.70E-05
miR-34c-5p	1.57	1.96E-03				miR-378b	−1.62	1.32E-04
miR-34b-5p	1.71	2.57E-03				miR-378c	−1.47	4.53E-04
miR-887	1.34	3.31E-03				miR-193a-5p	−1.32	2.22E-03
miR-182-5p	−1.01	3.82E-03				miR-320c	−1.45	3.48E-03
miR-135a-5p^3^	1.66	4.84E-03				miR-1285-3p	−1.25	6.90E-03
miR-184^2,3^	1.43	4.84E-03				miR-550a-3-5p	−1.09	1.36E-02
miR-30c-2-3p^3^	1.22	4.84E-03				miR-874	−1.06	1.39E-02
miR-873-3p	1.3	4.84E-03				miR-125a-5p	−1.01	1.69E-02
miR-125a-3p^3^	1.27	9.65E-03				miR-21-5p	0.98	1.69E-02
miR-671-3p	1.21	9.65E-03				miR-671-3p	−1.19	1.69E-02
miR-21-5p	−0.82	2.35E-02				let-7i-3p	−0.86	2.77E-02
miR-1285-3p	1.03	2.45E-02						
miR-375	−0.94	3.26E-02						
miR-3176	1.06	3.35E-02						
miR-127-3p^2^	0.92	3.74E-02						

Sample size for serum consisted of AD (n = 53), PD (n = 50) and Control (n = 62) subjects. Results were filtered at corrected p-value <0.05. We show only significant differentially expressed miRNAs with average number of mapped reads greater than 5 and 0.7< FC(log2) or FC(log2) <−0.7. Superscript 2 indicates differentially expressed miRNAs in both CSF and SER for the corresponding analysis. Superscript 3 indicates significant low abundance miRNAs with normalized base mean <10 mapped reads.

We also examined miRNAs that were different between AD and PD patients (**Table 1**;**Table 2**). In the CSF, only 1 of the 5 differentially expressed miRNAs between AD and PD subjects was specific to that analysis, and did not overlap with miRNAs that were detectably different in AD compared with control subjects or PD compared with control subjects: 32-5p. In SER, 16 miRNAs had different expression levels when AD and PD subjects were compared, out of which 12 were unique to that analysis and exhibited no overlap with results from CSF with AD or PD compared with control subjects.

### *miRNAs are differentially expressed in CSF and SER of PD patients*

In contrast to AD, only a handful of miRNAs have been identified as misregulated in PD patients by prior studies. A total of eight PD CSF samples and ten PD SER samples were removed prior to testing for differential expression due to low sample read count. Seventeen miRNAs were detected as significantly different at corrected p <0.05 between PD CSF (n = 57) and Control CSF (n = 65) samples (**Table 1**). Of the 17 miRNAs, 6 (∼35%) were previously identified to be differentially expressed in PD patients: let-7, 128, 433, 485-5p, 132, 212 [1], [32], [33], [34], [35], [36], [37], [38], [39], [40], [41], [42], [70]. Interestingly, miR-127-3p, 443, 431-3p, 136-3p and 10a-5p were differentially expressed for both AD compared to Control subjects and PD patients compared with Control subjects, in the CSF.

There were 5 miRNAs differentially expressed in SER samples from PD patients compared to control subjects. The expression levels of miR-338-3p, 30e-3p and 30a-3p were up-regulated in the serum of PD (n = 50) subjects, whereas miR-16-2-3p and 1294 were significantly down-regulated (**Table 2**). Of the 5 miRNAs, 16-2-3p, 30e, and 30a-3p (∼60%) were previously identified to be differentially expressed in Parkinson's subjects when compared to controls subjects [39], [43].

### *Potential novel miRNAs detected in CSF and SER*

We used miRDeep2 to predict novel miRNAs in our CSF and SER data [18], [44]. MiRDeep2 first aligns miRNA reads to the genomic reference, then uses an RNA fold tool to predict the RNA secondary structures in the sequence surrounding the aligned miRNA read and evaluates the structure and signature of each potential miRNA precursor. If the structure creates a miRNA hairpin and the potential miRNA read falls within the hairpin, as would be expected from Dicer processing, then the potential miRNA is assigned a score that reflects the calculated confidence in the predicted miRNA [45]. We used the following cutoffs: the miRNA must be expressed in at least 30% of either CSF samples or SER samples and expressed on average more than 5 times in each sample. Using these criteria, we detected a total of 13 novel miRNAs (**Table 3**). When we examined these new miRNAs for differential expression, only one displayed significant expression level changes between AD and PD SER samples at p <0.05 (statistical tests were corrected for multiple testing using all known plus potential miRNAs). The significant miRNA sequence is labeled bold in Table 3.

**Table 3:** Novel miRNAs in CSF and SER predicted by miRDeep2.

Mature Precursor Sequence	% of Serum Samples	% of CSF Samples	% of Total Samples
			
aguugggagagcauuagacuga_uuucuuuuuuucucuuucuga	21.94	62.07	42.36
**aggggccgagggagcgaga_gagcucugcggcgccaag**	36.22	35.47	35.84
ccaucugugggauuaugacuga_agucagaaucccacucaggug	12.76	44.33	28.82
uuuucgcucggccugggac_cucuggcccagggugguaugu	26.53	30.54	28.57
agguagauagaacaggucuugu_agaccuacuuaucuaccaaca	20.92	35.47	28.32
guaguggugguucagugg_agugcacaucuacag	9.18	33.99	21.8
ggggauguagcucagugguaga_ggccccggguucgauccccgg	8.67	32.02	20.55
ggaauugugguucagugg_auugaaccacaacuucuc	4.08	35.47	20.05
ucggcuguguaucucugugcc_cacagcguggcacagucgcgc	39.8	5.42	22.31
uugaggucggacaugguggcu_ccaccacgccuggccuaagagu	36.73	4.43	20.3
aggauuucugggcuguagugcgu_accuguggucccagcuccaug	32.65	1.97	17.04
cauggguacuggccugaaguc_uguugggacaagucugguggu	33.16	0.49	16.54
ccugggucugacacucuga_agggugcuggguuauuuccugggg	31.12	1.48	16.04

To be listed, the potential miRNA had to be present in at least 30% of either the SER or the CSF samples, and have more than 5 counts on average across all samples. Column one contains the precursor sequence predicted by miRDeep2 for the potential mature miRNA detected. Column two is the percentage of serum samples in which the miRNA was present (total number of serum samples examined: 196). Column three is the percentage of CSF samples in which the miRNA was detected (total number of CSF samples examined: 203). Column four represents the total percentage of samples in which the miRNA was detected.

### *miRNA expression in connection with Braak neurofibrillary stages, neurofibrillary tangle scores, and plaque-density scores*

We sought to investigate the correlation between miRNA expression data and the severity of pathology findings quantified at autopsy, regardless of disease diagnosis. We examined miRNAs that consistently increased or decreased their expression as measures of pathology increased. Ordinal logistic regression (OLR) was used to model the relationship between normalized miRNA counts and several ordinal outcome variables comprised of: i) Braak neurofibrillary stages; ii) neurofibrillary tangle scores and iii) plaque-density scores. Consequently, OLR was used for identification of miRNA markers associated with the progression of regional and time-dependent characteristics typical for AD pathology. Neuropathology examination at autopsy provided total Braak stages (1–6), neurofibrillary tangle scores (0–15) and plaque-density scores (1–15). The plaque and tangle scores were sums of pathology (0 =  none, 1 =  sparse, 2 =  moderate, 3 =  frequent) across five brain regions (Frontal, Temporal, Parietal, Hippocampal, Entorhinal). For additional information on patient scores, see **Table S1**. Prior to the analysis, neurofibrillary tangle and plaque-density scores were binned into 3 ordered response categories, with 1<2<3 for increasing gravity of progression. Similarly, Braak neurofibrillary stages were treated as ordinal under the assumption that levels of Braak staging have a natural stage ordering (1<2<3<4<5<6), with an unknown distance between adjacent levels. Upon filtering, each analysis consisted of the following number of subjects in each subgroup:

1. Braak stages: 1 (CSF n = 21, SER n = 21), 2 (CSF n = 21, SER n = 27), 3 (CSF n = 58, SER n = 44), 4 (CSF n = 37, SER n = 31), 5 (CSF n = 22, SER n = 23) and 6 (CSF n = 25, SER n = 18).
2. Neurofibrillary tangle stages: 1 (CSF n = 73, SER n = 71), 2 (CSF n = 58, SER n = 49) and 3 (CSF n = 53, SER n = 44).
3. Plaque-density stages: 1 (CSF n = 58, SER n = 55), 2 (CSF n = 41, SER n = 35), 3 (CSF n = 85, SER n = 74).

Ordinal logistic regression analysis resulted in several predictor variables (miRNAs) significant at unadjusted p- value <0.05, that consistently increased or decreased their expression across pathologic severity. We report miRNAs with the lowest Akaike Information Criterion (AIC) value, at the delta AIC <10 cut off (**Table 4** **,** **5** **,** **6**). For the reported models, parameter estimate 95% confidence interval did not include zero and data satisfied assumptions of the OLR.

**Table 4:** Braak neurofibrillary stage specific ordinal regression analysis of miRNA expression data.

Braak Stage CSF					Braak Stage SER				
has-mirName	Parameter Estimate	AIC	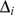	p-Value*	has-mirName	Parameter Estimate	AIC	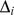	p-Value*
miR-9-3p	−9.60E-03	621.05	0	2.31E-04	let-7i-3p	1.50E-02	571.21	0	3.39E-04
miR-181a-5p	−3.83E-05	623.51	2.46	6.10E-04	miR-1307-5p	5.71E-04	576.26	5.05	1.59E-02
miR-181a-3p	−7.27E-03	626.36	5.31	2.38E-03	miR-183b-5p	−2.66E-03	576.33	5.12	4.13E-03
miR-760	−2.11E-02	627.15	6.09	3.99E-03	miR-1285-3p	2.43E-03	576.44	5.23	5.90E-03
miR-136-3p	−4.24E-03	627.19	6.14	3.93E-03	miR-3176	9.98E-03	577.72	6.51	1.65E-02
miR-421	−6.18E-03	627.67	6.61	5.50E-03	miR-30c-3p	2.47E-02	578.86	7.65	3.27E-02
miR-105-5p	1.35E-02	627.67	6.62	6.40E-03	miR-16-5p	−6.08E-05	579.03	7.82	2.00E-02
miR-769-5p	−2.73E-04	628.06	7.01	7.81E-03	miR-3615	6.53E-04	579.22	8.01	2.05E-02
miR-181b-5p	−2.23E-04	628.49	7.43	1.14E-02	miR-671-3p	3.26E-03	579.24	8.03	2.48E-02
miR-181d	−2.68E-04	629.25	8.2	1.72E-02	miR-93-5p	−8.36E-04	579.76	8.55	2.51E-02
miR-664-3p	3.66E-03	629.64	8.58	1.24E-02	miR-200a-3p	−1.89E-03	579.83	8.62	2.59E-02
miR-330-3p	−2.22E-02	629.92	8.87	1.75E-02	miR-155-5p	−9.85E-03	579.84	8.63	2.62E-02
miR-329	−3.19E-02	630.03	8.97	1.86E-02	miR-181c-3p	3.67E-03	580.03	8.82	4.46E-02
miR-539-3p	−6.62E-02	630.14	9.08	1.92E-02	miR-146b-5p	−2.13E-04	580.22	9.01	3.32E-02
miR-431-3p	−4.12E-02	630.14	9.09	2.09E-02	miR-125b-5p	7.71E-04	580.38	9.17	3.76E-02
miR-132-3p	−7.30E-03	630.29	9.24	2.70E-02					
miR-574-3p	1.27E-03	630.8	9.75	4.76E-02					
miR-708-3p	−1.31E-02	630.96	9.91	3.53E-02					

Ordinal logistic regression analysis (OLR) was implemented in order to detect miRNAs with monotonic expression patterns across Braak neurofibrillary stages. Braak stages were recorded during autopsy for each subject, and specific CSF subgroups consisted of stage 1 (n = 21), stage 2 (n = 21), stage 3 (n = 58), stage 4 (n = 37), stage 5 (n = 22), and stage 6 (n = 25) samples. For SER, Braak subcategories comprised stage 1 (n = 21), stage 2 (n = 27), stage 3 (n = 44), stage 4 (n = 31), stage 5 (n = 23), and stage 6 (n = 18) samples. Delta AIC (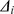) quantifies the information loss associated with using each model relative to the best approximating model. We report predictor variables with the lowest Akaike Information Criterion (AIC) and 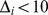 that satisfy assumptions of the OLR. p-Value* is unadjusted.

**Table 5:** miRNAs associated with neurofibrillary tangle score.

Tangles CSF					Tangles SER				
has-mirName	Parameter Estimate	AIC	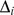	p-Value*	has-mirName	Parameter Estimate	AIC	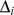	p-Value*
miR-9-3p	−9.00E-03	387.84	0	2.66E-03	miR-429	−2.14E-01	335.95	0	7.21E-03
miR-421	−8.12E-03	389.01	1.16	4.03E-03	let-7i-3p	1.78E-02	337	1.05	8.86E-04
miR-760	−2.24E-02	390.32	2.47	8.45E-03	miR-21-5p	−1.23E-04	339.81	3.86	3.23E-03
miR-181d	−3.32E-04	390.37	2.53	1.19E-02	miR-141-3p	−3.71E-03	343.24	7.3	3.45E-02
miR-181b-5p	−2.52E-04	390.61	2.77	1.17E-02	miR-200a-3p	−4.08E-03	343.29	7.35	3.65E-02
miR-184	−1.56E-02	391.52	3.67	3.27E-02	miR-3176	1.35E-02	343.53	7.58	2.67E-02
miR-127	−1.39E-02	391.53	3.68	2.46E-02	miR-374b-5p	−2.69E-02	344.04	8.09	1.99E-02
miR-129-5p	−1.15E-03	391.83	3.99	2.50E-02	miR-183-5p	−3.49E-03	344.56	8.61	1.45E-02
miR-148b-5p	4.91E-02	391.89	4.04	1.21E-02	miR-301a-3p	−2.00E-02	345.23	9.29	2.00E-02
miR-181a-5p	−5.75E-03	392.54	4.7	2.30E-02	miR-10a-5p	−2.09E-05	345.24	9.29	2.04E-02
miR-499a-5p	−1.14E-01	392.75	4.9	2.40E-02	miR-17-3p	7.61E-03	345.48	9.54	2.59E-02
miR-330-3p	−2.22E-02	393.38	5.53	2.88E-02	miR-432-5p	−9.25E-03	345.86	9.92	2.90E-02
miR-219-3p	−6.05E-04	393.42	5.58	4.05E-02					
miR-592	2.74E-03	393.77	5.93	3.18E-02					
miR-101-5p	−4.09E-02	394.02	6.17	4.11E-02					
miR-708-3p	−1.37E-02	394.16	6.31	4.90E-02					
miR-30b-5p	6.57E-04	394.3	6.45	4.39E-02					
miR-30c-5p	3.00E-04	394.42	6.58	4.61E-02					

Neuropathological examination disclosed total neurofibrillary tangele scores. We binned the data 0–15, in increasing increments, for each subject. Scores were divided into three groups corresponding to low neurofibrillary tangeles score (0–4), moderate neurofibrillary tangeles score (5–9) and high neurofibrillary tangeles score (10–15). Ultimately, neurofibrillary tangle subgroups consisted of stage 1 (n = 73), stage 2 (n = 58) and stage 3 (n = 53) subjects for CSF and stage 1 (n = 71), stage 2 (n = 49) and stage 3 (n = 44) for SER. Ordinal logistic regression analysis (OLR) was implemented in order to fit miRNA expression data across the three ordered groups. Delta AIC 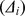 quantifies the information loss associated with using each model relative to the best approximating model. We report predictor variables with the lowest Akaike Information Criterion (AIC) and 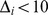 that satisfy assumptions of the OLR. p-Value* is unadjusted.

**Table 6:** miRNAs associated with plaque density score.

Plaques CSF					Plaques SER				
has-mirName	Parameter Estimate	AIC	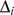	p-Value*	has-mirName	Parameter Estimate	AIC	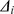	p-Value*
miR-184	−2.72E-02	375.68	0	8.25E-04	miR-30b-5p	−5.13E-03	342.46	1.14	2.35E-02
miR-335-5p	−2.02E-03	380.8	5.13	4.23E-03	miR-183-5p	−3.39E-03	342.58	1.26	9.81E-03
miR-199b-5p	−5.85E-02	380.82	5.15	9.97E-03	miR-106a-5p	−1.13E-02	343.33	2.01	1.95E-02
miR-760	−2.42E-02	381.91	6.23	4.32E-03	miR-339-3p	−4.98E-03	344.19	2.87	2.78E-02
miR-1299	−3.29E-02	382.46	6.78	1.15E-02	miR-625-3p	−7.96E-02	344.23	2.91	2.88E-02
miR-455-5p	−6.20E-02	382.73	7.05	1.29E-02	miR-17-5p	−7.93E-03	345	3.69	3.74E-02
miR-708-3p	−1.96E-02	383.14	7.46	8.94E-03	miR-93-5p	−8.50E-04	345.53	4.22	4.03E-02
miR-125b-3p	−1.14E-03	383.63	7.95	9.94E-03					
miR-376a-3p	−7.24E-02	384.32	8.65	1.19E-02					
miR-195-5p	−9.46E-04	384.47	8.79	1.86E-02					
miR-548b-5p	−8.07E-02	384.5	8.83	1.18E-02					
miR-101-5p	−5.23E-02	384.76	9.09	1.40E-02					
miR-549	−2.33E-02	384.85	9.17	3.01E-02					
miR-651	−8.13E-02	384.9	9.22	1.65E-02					
miR-19b-3p	−6.33E-04	385.06	9.38	3.33E-02					
miR-19a-3p	−8.25E-04	385.09	9.41	3.19E-02					
miR-101-3p	−1.28E-04	385.21	9.54	3.31E-02					

Neuropathological examination disclosed total plaque-density score ranging from 1–15 for each subject. Scores were divided into three groups corresponding to low plaque-density score (1–5), moderate plaque-density score (6–10) and high plaque-density score (11–15). Ultimately, plaque density subgroups consisted of stage 1 (n = 58), stage 2 (n = 41) and stage 3 (n = 85) subjects for CSF and stage 1 (n = 55), stage 2 (n = 35) and stage 3 (n = 74) for SER. The ordinal regression method was used to model the relationship between the ordinal outcome variable, plaque density score, and normalized miRNA counts as explanatory variable. Delta AIC 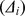 quantifies the information loss associated with using each model relative to the best approximating model. We report miRNAs with the lowest AIC value and 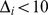. p-Value* is unadjusted.

**Figure 2:**
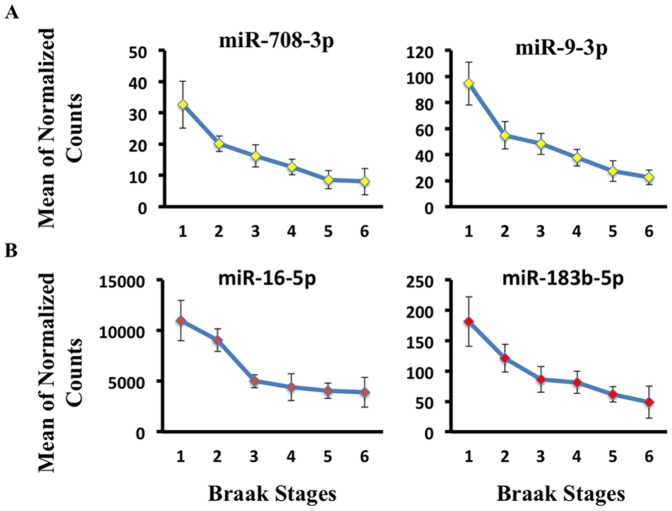
Ordinal regression analysis reveals miRNAs with progressive expression trends across increasing Braak stages. (**A**) We plotted two miRNAs selected from Table? 4 (miR-9-3p and miR-708-3p) that are detected in CSF and change with increasing Braak stage. The y axis is the mean of normalized counts for each miRNA, while the x axis represents Braak stages. (**B**) miR-16-5p and miR-183b-5p are detected in SER and change with Braak stage.

**Figure 3:**
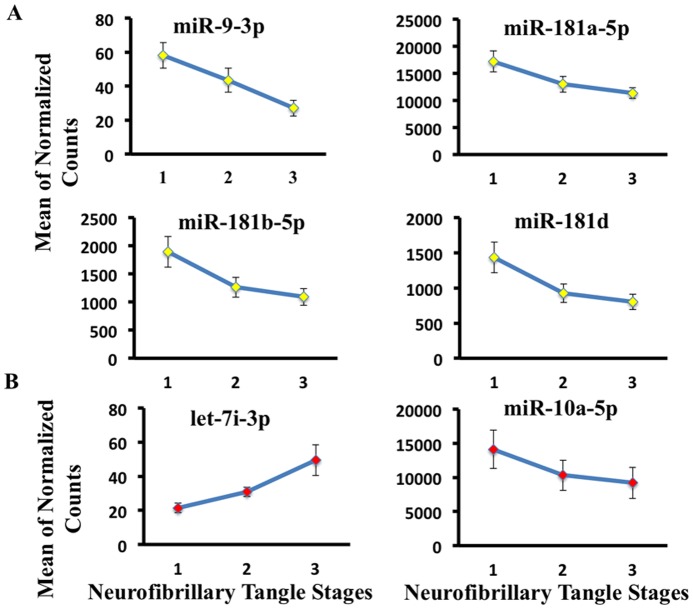
Ordinal regression analysis reveals miRNAs with progressive expression trends across increasing neurofibrillary tangle density. (**A**) We plotted four miRNAs (miR-181b-5p, miR-181d, miR-181a-5p and miR-9-3p) detected in CSF from Table? 5. (**B**) miR-7i-3p and miR-10a-5p were selected from Table? 5, significant for neurofibrillary tangle stage regression analysis in SER.

**Figure 4:**
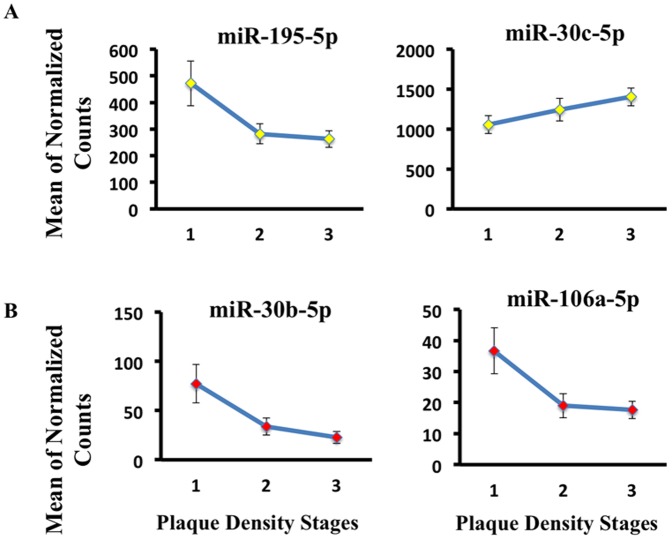
Ordinal regression analysis reveals miRNAs with progressive expression trends across increasing amyloid plaque density. (**A**) We plotted two miRNAs (miR-195-5p, miR-101-3p) detected in CSF from Table? 6 that showed consistent expression changes with increased density of plaques. (**B**) miR-106-5p and miR-30b-5p, detected in SER and selected from Table? 6, showed significant fit across increasing plaque density stages.

i)
  - CSF Braak stages: 18 miRNAs, including miR-9-3p and miR-708-3p (**Table 4** **,** **Figure 2A** **)**. We plotted two miRNAs selected from Table 4 (miR-9-3p and miR-708-3p) that are detected in CSF and change with increasing Braak stage. The y axis is the mean of normalized counts for each miRNA, while the x axis represents Braak stages.
  - SER Braak stages: 15 miRNAs including miR-16-5p and miR-183b-5p (**Table 4** **,** **Figure 2B**). miR-16-5p and miR-183b-5p are detected in SER and change with Braak stage.
ii)
  - CSF neurofibrillary tangle stages: Neuropathology examination disclosed total neurofibrillary tangle scores. Scores were created by counting tangle pathology (0 =  none, 1 =  sparse, 2 =  moderate, 3 =  frequent) across several brain regions (Frontal, Temporal, Parietal, Hippocampal, Entorhinal) (**Table S1**). We binned the data 0–15, in increasing increments, for each subject. Summed total scores were divided into three groups corresponding to low neurofibrillary tangles score (0–4), moderate neurofibrillary tangles score (5–9) and high neurofibrillary tangles score (10–15). Ordinal regression analysis was implemented in order to fit miRNA expression data across the three ordered groups. We report miRNAs with the lowest Akaike Information Criterion (AIC), significant at uncorrected p-value <0.05 cut off if the parameter estimate 95% confidence interval did not include zero. The ordinal logistic regression analysis resulted in 18 reported miRNAs including miR-9-3p and the miR-181 family (**Table 5** **,** **Figure 3A**). We plotted four miRNAs (miR-181b-5p, miR-181d, miR-181a-5p and miR-9-3p) detected in CSF from Table 5 with delta AIC <10.
  - SER neurofibrillary tangle stage: 12 reported miRNAs including let-7i-3p and miR-10a-5p (**Table 5** **,** **Figure 3B**). let-7i-3p and miR-10a-5p were selected from Table 5, significant for neurofibrillary tangle stage regression analysis in SER.
iii)
  - CSF plaque-density stages: Neuropathology characterization of total plaque-density scores, ranging from 1–15 for each subject. Scores were summed from five brain regions described above. Total scores were divided into three groups corresponding to low plaque-density score (1–5), moderate plaque-density score (6–10) and high plaque-density score (11–15). The ordinal regression method was used to model the relationship between the ordinal outcome variable, plaque density score, and normalized miRNA counts as explanatory variable. We report miRNAs with the lowest AIC significant at uncorrected p-value <0.05 if the parameter estimate 95% confidence interval does not include zero. We plotted two miRNAs out of the 17 reported (miR-195-5p, miR-101-3p) in Table 6 that showed consistent expression changes with increased density of plaques (**Table 6** **,** **Figure 4A**).
  - SER plaque-density stages: 7 miRNAs including miR-106a-5p and miR-30b-5p (**Table 6** **,** **Figure 4B**). miR-106-5p and miR-30b-5p, detected in SER and selected from Table 6, showed significant fit across increasing plaque density stages.

### *miRNA expression correlated with substantia nigra depigmentation and Lewy body pathology*

**Figure 5:**
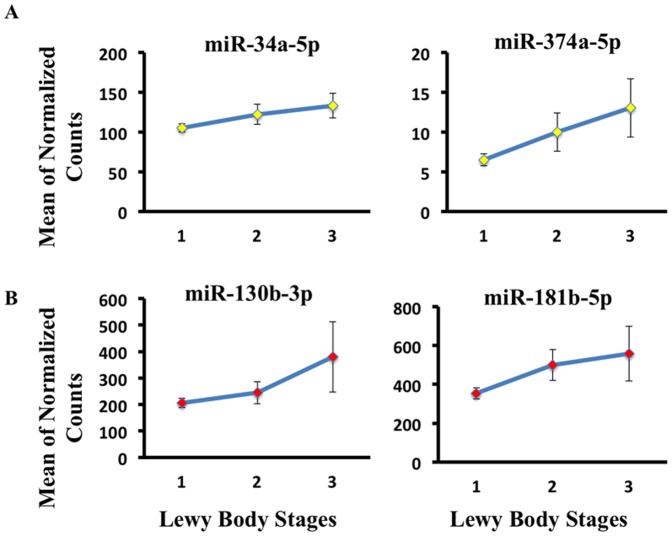
Ordinal regression analysis reveals miRNAs with trends in Lewy body progression. (**A**) We plotted two miRNAs (miR-34a-5p and miR-374-5p) detected in CSF from Table? 7 that showed consistent expression change with progression of Lewy bodies. (**B**) We plotted two miRNAs (miR-130b-3p and miR-181b-5p) detected in SER from Table? 7 that showed consistent expression changes with progression of Lewy bodies.

The progressive loss of of melanin-containing dopaminergic neurons in the substantia nigra leads to a loss of pigmentation, resulting in measurable depletion of staining in the tissue. The depigmentation score correlates well with the loss of striatal tyrosine hydroxylase reactivity. For the subjects in this study, depigmentation pathology was assessed according to Beach et al., 2009 [46]. No differentially expressed miRNAs were detected from comparing moderate and severe depigmentation in samples with Limbic type Lewy body progression. The spread of Lewy bodies and Lewy neurites from the brainstem to the cerebral cortex is one of the best correlations of PD progression to PD with dementia (PDD) [46], [47], [48]. Olfactory bulb and tract, brainstem IX–X, brainstem (locus coeruleus), brainstem (substantia nigra), amygdala, transentorhinal, anterior cingulate gyrus and neocortex (temporal, frontal and parietal) were assessed via histopathology to calculate the Lewy-related density scores for aggregate formation with all immunoreactive features in the regions noted (the antibody used was against phosphorylated 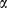 -synuclein) [46]. Neuronal perikaryal cytoplasmic staining, neurites and puncta are all considered together, using the templates provided by the Dementia with Lewy Bodies Consortium [49]. Scores are binned from 0–2, 0 being no Lewy body detection to 2 being the highest (neocortical type). Upon filtering, OLR analysis consisted of the following number of subjects in each subgroup: no Lewy bodies (CSF: n = 126; SER: n = 113), Limbic type (CSF: n = 30; SER: n = 23) and Neocortical type (CSF: n = 21; SER: n = 20). Total of 12 miRNAs in CSF and 10 in SER were reported as best singular predictor models of Lewy body stage progression (**Table 7**). Normalized read counts for miR34a-5p and miR-374a-5p are displayed in **Figure 5**. Interestingly, our OLR results indicate that miR-132 expression monotonically decreases in CSF as Lewy body pathology advances- findings concurrent with decreased expression levels of miR-132 in PD samples compared to controls (**Table1;** **Table 7**).

**Table 7:** Lewy body progression-associated miRNAs.

Lewy Body CSF					Lewy Body SER				
has-mirName	Parameter Estimate	AIC	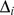	p-Value*	has-mirName	Parameter Estimate	AIC	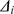	p-Value*
miR-34a-3p	1.26E-01	277.83	0	2.38E-03	miR-28-3p	4.04E-04	242.15	0	1.17E-02
miR-132-5p	−1.55E-01	278.04	0.21	1.54E-02	miR-199a-5p	4.46E-03	244.31	2.16	2.60E-02
miR-374a-5p	3.61E-02	280.11	2.28	8.64E-03	miR-181b-5p	9.72E-04	244.33	2.18	2.58E-02
miR-19a-3p	6.99E-04	280.33	2.5	1.83E-02	miR-145-5p	5.56E-03	244.49	2.33	3.23E-02
miR-19b-3p	5.03E-04	280.8	2.97	2.45E-02	miR-181d	1.27E-03	244.53	2.38	2.92E-02
miR-28-5p	−1.10E-02	281.74	3.91	2.75E-02	miR-5588-5p	3.77E-02	244.96	2.81	3.66E-02
miR-3613-5p	8.77E-02	281.78	3.95	2.43E-02	miR-548w	9.49E-02	244.99	2.84	4.01E-02
miR-29a-3p	6.97E-04	282.08	4.26	2.61E-02	miR-27b-5p	3.06E-02	245.07	2.92	4.46E-02
miR-29c-3p	6.88E-04	282.3	4.48	3.06E-02	miR-425-3p	1.05E-02	245.28	3.13	4.72E-02
miR-34a-5p	5.28E-03	282.53	4.71	3.18E-02	miR-130b-3p	1.18E-03	245.29	3.14	4.60E-02
miR-542-3p	4.57E-02	282.77	4.95	4.02E-02					
miR-944	6.90E-02	283.07	5.24	4.64E-02					

Ordinal regression analysis was implemented in order to detect miRNAs with monotonic expression patterns across Lewy body stages. Lewy body stages were defined with the Unified Staging System for Lewy Body Disorders as described by Beach et al [46]. Specific CSF Lewy body stage subgroups consisted of: no Lewy bodies (n = 126), Limbic type (n = 30) and Neocortical type (n = 21). Similarly, Lewy body subcategories in the SER were comprised of: no Lewy bodies (n = 113), Limbic type (n = 23) and Neocortical type (n = 20). We report predictor variables with the lowest Akaike Information Criterion (AIC) and 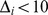 that satisfy assumptions of the OLR. p-Value* is unadjusted.

### *miRNA expression, potential markers of cognition*

Thirty-four miRNAs had significant differential expression in serum samples when comparing PD patients with PD with a clinical diagnosis of dementia (PDD) (**Table S3**). We were interested to know whether or not these same PDD miRNAs were significantly different in our serum data from AD patients compared to normal controls. We found that 3 out of the 34 miRNAs had significantly altered expression in AD subjects as well (**Table 8**). Sample size for serum consisted of PD (n = 32), PDD (n = 18), AD (n = 53) and Control (n = 62) subjects. Results were filtered at corrected p-value <0.05, and the logarithmic base 2 fold change (FC) is relative to the first listed group for each comparison.

**Table 8:** miRNAs significantly different in SER samples from PD vs. PDD and Control vs. AD.

Parkinson's vs Parkinson's with Dementia			Control subjects vs Alzheimer's subjects		
hsa-mirName	FC (log2)	Adjusted p-Value	hsa-mirName	FC (log2)	Adjusted p-Value
miR-34c-5p	2.12	0.002	miR-34c-5p	1.57	0.002
miR-34b-5p	2.01	0.009	miR-34b-5p	1.71	0.003
miR-375	−1.61	0.008	miR-375	−0.94	0.033

Sample size for serum consisted of PD (n = 322), PDD (n = 188), AD (n = 53) and Control (n = 62) subjects. Results were filtered at corrected p-value <0.05. The logarithmic base 2 fold change (FC) is relative to the first listed group for each comparison. P-Values are adjusted for multiple corrections.

One of the differentially expressed miRNAs, miR-34c, was previously identified to be highly expressed in the hippocampus of patients with AD and in animal models of AD [50]. The same group linked miR-34c as a negative regulator of memory consolidation [50]. Interestingly, our data examining miRNAs differentially expressed in the progression of Lewy bodies from limbic to neocortical, also identified miR-34c and 34b as significantly altered. While we identified miRNAs detectible in blood (serum) that have the potential to indicate cognitive impairment, CSF had revealed only 11 significant differentially expressed miRNAs and no overlap with the AD and Control CSF analysis.

## Discussion

These data represent one of the largest data sets to date, examining the miRNAs detectable in cell-free biofluids from patients with neurodegenerative disease, and the first to use NGS to compare the profiles from CSF and SER. We were able to detect differentially expressed miRNAs in CSF and SER, many of them previously identified to be misregulated in patient tissue samples. Interestingly, there was minimal overlap between the miRNAs identified in CSF with the miRNAs identified in SER. Further temporal investigation in a living cohort will be necessary to determine which biofluid will be most reliable for early detection of disease and predictive of disease progression. These data are an important first step, comparing the biofluid profiles with one another and with known miRNAs deregulated in brain tissue. The examination of miRNA changes associated with the severity of disease pathology can also provide important insight about how to interpret miRNA changes as diagnostic and prognostic indicators of disease.

### *miRNAs of particular interest*

Many of the miRNAs we were able to detect as differentially expressed in cell-free CSF and SER have been reported previously in studies examining brain tissue from patients with AD and PD [22], [24], [26], [29], [51], [52], [53], [54], or as miRNAs that target genes of particular interest- such as APP, BACE1 and α-synuclein [54], [55], [56]. For example, 73% of the miRNAs we identified to be differentially expressed in AD patient CSF compared with control subject CSF were previously found to be deregulated in AD brain tissue or target known AD-related mRNAs. We selected a few of the differentially expressed miRNAs for further discussion.

We found **miRNA-9** to be downregulated in CSF from AD patients when compared to levels in CSF from control subjects. miRNA-9 expression levels change across Braak stages and neurofibrillary tangle advancement in CSF, decreasing with Alzheimer's disease progression. To date, several studies demonstrate the altered expression of miR-9 in AD brains [22], [23], [55], [57]. The gene coding for neurofilament H is among the miR-9 targets potentially involved in AD [58]. This protein has previously been shown to be upregulated in disease conditions and can be isolated from NFTs along with Tau and other cytoskeleton proteins [58], [59], [60], [61]. These observations correlate with the decrease in miR-9 levels we observed with tangle severity. In addition, miR-9 has been shown to be downregulated in response to 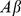 treatment in primary neurons, suggesting that miR-9 downregulation could be a consequence of the disease pathogenesis that results in neurofilament-H upregulation [2]. However, miR-9 also targets Sirtuin (SIRT1), a de-acetylase with reduced expression in AD brains [62], [63]. In contrast to neurofilament H, decreased SIRT1 levels would indicate a potential increase in miR-9, or the increase of another miRNA targeting SIRT1. Interestingly, SIRT1 can also be regulated by miR-34c (below).

**miR-34c** was found in our study to be upregulated in PDD patients compared with PD patients and in AD patients compared to control subjects. Zovoilis et al. [50] found high levels of miR-34c in hippocampus of AD patients and in animal models of AD. They observed that, when miR-34c is elevated, memory consolidation is impaired. When miR-34c is targeted for removal, learning and memory is restored. One of the mRNA targets for miR-34c is SIRT1, involved in synaptic plasticity and memory formation [64]. The authors confirmed that elevated miR-34c correlated with decrease in SIRT1 in tissue samples. The authors did not look for the expression of any miRNAs in AD patient blood samples, nor did they examine PD or PDD patients. The hypothesis that elevated levels of miR-34c is related to cognitive decline holds true in our data from patient serum samples. There is approximately a 2.1-log2 fold increase in miR-34c in PDD patient serum compared with PD patients and a 1.6-log2 fold increase in miR-34c in AD patient serum compared with normal control subjects.

**miR-34b/c** is also associated with PD. Levels of miR-34b/c are decreased by 40–65% in amygdala, substantia nigra, cerebellum and frontal cortex of PD patients [33]. Additionally, knock-down of miR-34b/c in differentiated SH-SY5Y neuroblastoma cells resulted in a decrease in parkin and DJ-1 (encoded by PARK7) concentrations that led to a disturbance of mitochondria function and decrease in viability of the cell [65]. DJ-1 can be involved in regulation of apoptosis; it can also act as a redox chaperone inhibiting the aggregation of 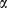 -synuclein [66]. Cell death associated with altered mitochondrial activity and oxidative stress are recognized biochemical abnormalities associated with PD. It remains to be proven whether the decreased expression of these miRNAs is due to their specific down-regulation in surviving neurons or secondary to neuron degeneration.

**miR-101** was decreased in CSF, and correlated with increases in neurofibrillary tangles and plaque density. Several independent studies showed that miR-101 was downregulated in human AD cortex [23], [57], [67]. Cyclooxydenase-2 (COX-2) and APP are known miR-101 targets implicated in AD [15]. COX-2 is involved in the inflammatory response, associated with neuronal loss, colocalizes with NFTs, and is deregulated in the AD brain [15], [67]. It is possible that miR-101 down-regulation might contribute significantly to AD pathology by: 1) increasing APP expression; 2) promoting NFT formation through the increase in Tau phosphorylation; 3) contributing to inflammation through the upregulation of COX-2 expression.

Expression of **miR-132** has been previously described as required for neuron morphogenesis and function, whereas significant down-regulation in miR-132 expression has been associated with 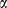 -synuclein accumulation and neuronal malfunction in 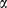 -synuclein (A30P)-transgenic mice [68], [69]. Yang et al. demonstrated through bioinformatics prediction, luciferase-reporter assay, and Western blot analysis that miR-132 could directly regulate expression of Nurr1, a critical transcription factor for midbrain dopamine neuron development and differentiation [70]. Additionally, Yang et al. showed that inhibition of endogenous miR-132 significantly increases differentiation of dopamine neurons, whereas prolific expression of miR-132 in embryonic stem cells dramatically represses dopamine neuron differentiation with no effect on the total number of neurons [70]. As a potential regulator of methyl-CpG-binding protein, an important component of neurodevelopment and neurodegeneration, miR-132 is a prospective molecule of interest in PD diagnosis and treatment [70].

## Conclusion

One of the first decisions most researchers studying markers of neurodegeneration must consider before they begin a project is what tissue or biofluid to profile. We provide a comprehensive examination of miRNAs detected in CSF and blood from the same patients and a comparison to reported miRNAs deregulated in brain tissue from AD and PD. In living patients, accessible tissue samples are limited. Among readily available biofluids, we can examine urine, saliva, and serum; CSF is more difficult to obtain. Although recently saliva and salivary gland biopsies have been shown to contain potential markers of PD, the utility of urine and saliva samples for profiling neurodegenerative disease or central nervous system damage still needs further examination [71]. For this study, we concentrated our analysis on CSF and serum from blood. CSF is in close proximity to the diseased tissue, but is often difficult to obtain from subjects. Blood is easier to acquire, but may not reliably reflect changes associated with neurodegeneration. When we compared the miRNA profiles from the two biofluids, we found that miRNAs detected in CSF cluster patients slightly more effectively than miRNAs detected in SER (Figures S1, S2). However, depending on individual analyses, there appeared to be benefits to both biofluids. For example, 73% of the deregulated miRNAs identified in our CSF data from AD patients were previously reported. However, comparison of miRNAs that overlap between PD with PDD and AD with cognitively normal controls revealed changes only in SER samples.

There are many more studies and data available for miRNA deregulation in association with AD than with PD. We found deregulated miRNAs associated with both diseases and present in both CSF and SER biofluids; interestingly, there were consistently fewer miRNAs associated with PD in each of the analyses we performed. There are several reasons why this may be the case: 1) patients with AD have significant ongoing spread of the disease from one brain region to another with severe plaque deposition and tangle pathologies. Perhaps these pathologies are more significant drivers of miRNA deregulation and detection, 2) patients with PD display mild to moderate plaque and tangle pathology in addition to Lewy bodies, leading to potentially fewer detected miRNAs specifically indicative of the disease, and 3) by the time of death, the destruction of several of the specific brain regions and cell types associated with PD (substantia nigra and striatum), have already occurred. PD patients begin to experience symptoms upon the loss of 50–60% of dopaminergic neurons within the substantia nigra, and severe depletion of dopamine in the striatum [1], [72]. This may contribute significantly to a reduction in detectable disease-related miRNAs late in the disease.

We will continue to evaluate many of the miRNAs identified in this paper using additional methods and samples. We will use qRT-PCR as an additional assay for validation of differentially expressed miRNAs as well as sequencing to validate the presence of miRNAs in SER from patients living with the disease, early in their diagnosis. We will also examine the possible enrichment of specific miRNAs within microvesicles or associated with extracellular RNA-binding proteins. Ultimately validation of these miRNAs in larger patient cohorts will enable the research community to identify the critical miRNA biomarkers that are most clearly associated with specific neurodegenerative disorders, stage and severity of disease.

## Materials and Methods

### *Samples and patient data*

Ethics Statement - All subjects were enrolled in the Banner Sun Health Research Institute (BSHRI) Brain and Body Donation Program as a whole-body donor and had previously signed informed consent approved by the BSHRI Institutional Review Board (IRB). The TGen Office of Research Compliance approved the use of the banked postmortem samples for this study. We obtained the following three groups of samples that were used for this study: AD (n = 67 CSF and n = 64 SER), PD (n = 65 CSF and n = 60 SER), and control (n = 70 CSF and n = 72 SER) from the Sun Health Research Institute, Sun City AZ. Verification of the diagnosis using neuropathology evaluations was completed and reported for all samples. A comprehensive overview of the cohort and data collected is included in Table S1. Figure S1 displays no significant source of variation in samples due to age, gender, or postmortem interval (PMI).

### *RNA isolation and sequencing*

Total RNA was isolated from 1ml of CSF and 1ml of SER from each subject as described in Burgos et al., 2013 [17]. Briefly, the miRVana PARIS kit (Invitrogen) was used with a modified protocol to extract total RNA and maximize miRNA yield. The Illumina TruSeq Small RNA sequencing kit was used for library preparation as previously described [17]. The samples were given individual barcodes up to 48, pooled and loaded on seven lanes of the Illumina HiSeq2000 with one lane of the flowcell used as a control for calculating phasing throughout the run. Each sample was often sequenced on two different flowcells to maximize reads mapped to mature miRNA sequences in miRBase.

### *Post-sequencing analysis pipeline*

Sequencing data generated by Illumina HiSeq2000 was pre-processed as previously described in Metpally et al., 2013 [44] and aligned to the reference with miRDeep2 software [45]. The sequencing data was processed and de-multiplexed using Illumina's CASAVA (v1.8) pipeline. Quality control checks on raw fastq reads generated by CASAVA were preformed by FastQC software. The FASTX toolkit was used for fastq pre-alignment processing, including adapter clipping and read collapsing, for better mapping results. Illumina three prime adapter sequences were removed by the fastx_clipper tool. Clipped reads were used as an input argument for miRDeep2 alignment software.

The processing of sequencing data using miRDeep2 consists of three modules. The Mapper module preforms read preprocessing and alignment to the reference genome. Once aligned, the miRDeep2 module excises genomic regions covered by the sequencing data in order to identify probable secondary RNA structure. Plausible miRNA precursors are evaluated and scored based on their likelihood of being true events. The Quantifier module produces a scored list of known and novel miRNAs with quantification and expression profiling. We used default parameters suggested by the creators of the tool and allowed one single nucleotide variation (SNV). The csv files from miRDeep2 were used for further analysis. All sequencing associated with the samples can be found with accession phs000727.v1.p1 in dbGaP.

### *Normalization and quality control*

The miRNA read counts identified by miRDeep2 were normalized using DESeq2 normalization method to account for compositional bias in sequenced libraries and library size. Assuming typical DESeq2 data frame, the method consists of computing a size factor for each sample as the median ratio of the read count over the corresponding row geometric average [73]. Raw counts were then divided by the size factor associated with their sample [73]. Under DESeq2 normalization hypothesis, most genes are not differentially expressed (DE), leading to a ratio of 1. Therefore, the size factor for the sample is an estimate of the correction factor that needs to be applied to all read counts of the corresponding column in order to make samples comparable.

Quality control of miRNA expression data consisted of filtering both samples and miRNAs. Samples with total sum of mapped read counts lower than 100,000 for CSF and 60,000 for SER were removed. Thresholds were determined based on the distribution of the total counts for all samples. Additionally, miRNAs with average less than 5 counts were not considered for further analysis.

### *Differential expression*

Differential expression of miRNA read counts was performed using DESeq2 (v2.1.0.19) package [74]. Three groups were considered for paired analysis from CSF data: i) Control and Alzheimer's subjects, ii) Control and Parkinson's subjects, and iii) Alzheimer's and Parkinson's subjects. Similarly, three groups were considered for paired analysis from SER data: i) Control and Alzheimer's subjects, ii) Control and Parkinson's subjects, and iii) Alzheimer's and Parkinson's subjects. DESeq2 method is based on negative binomial distribution (NB), with custom fit for variance-mean dependence [74]. Upon normalization, dispersion is estimated by local regression for gamma-family generalized linear models, providing basis for inference. Sum of all replicates for gene *i* corresponding to conditions *A* and *B*, 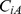 and 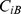, are evaluated as NB-distributed with moments as estimated and fitted. The p value of a pair of observed count sums 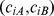 is then the sum of all probabilities less or equal to 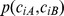, conditioned on 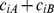 [74]. We report differentially expressed miRNA with fold change 0.7< FC(log2) or FC(log2) <−0.7 significant at adjusted p-value <0.05.

### *Regression analysis*

To take advantage of the ordinal nature of regional and time-depended characteristics present in AD and PD pathology, we implemented ordinal logistic regression (OLR) in order to detect miRNAs with monotonic expression patterns. The ordinal logistic model assumes the presence of a covert continuous predictor variable and ordinal outcome that arises from discretization of the underlying continuum into *j*-ordered groups such that j = [1…J] [75]. Analysis of ordered categorical data was executed via cumulative link models (CLMs). Ordinal response variable 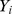 then follows multinomial distribution with probability 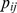 that the *i*th observation falls in response cathegory *j*. Ordinal logit considers the probability of a single event and all events that are ordered before it, hence incorporating ordered nature of the dependent variable in the fit [75]. With cumulative probabilities set 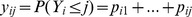, cumulative logits which incorporate the logit link are defined as:
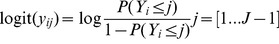
(1)Let 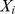 be a vector of explanatory variables, 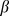 the corresponding set of regression parameters, and 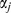 provides each cumulative logit its unique intercept value. Then, cumulative logit model is a regression model for cumulative logits defined as:
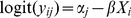
(2)Four well described signatures of AD and PD pathology were binned into ordinal categories and considered as OLR outcome variables: i) Braak neurofibrillary stages, ii) neurofibrillary tangle scores, iii) plaque-density scores and iv) synuclein/Lewy body stages. Neuropathology examination disclosed total Braak stages (1–6), neurofibrillary tangle neurofibrillary tangle (0–15), plaque-density scores (1–15) and Lewy body stages (no Lewy bodies; Limbic type; Neocortical type). For convenience, we binned the neurofibrillary tangle and plaque-density scores for each subject into three ordinal categories, in increasing increaments. The events of interest correspond to low neurofibrillary tangles score (0–4), moderate neurofibrillary tangles score (5–9) and high neurofibrillary tangles score (10–15). Similarly, for plaque-density data three groups correspond to low plaque-density score (1–5), moderate plaque-density score (6–10), and high plaque-density score (11–15). Lastly, synuclein/Lewy body stage was divided into ordinal outcome variables as defined by the Unified Staging System for Lewy Body Disorders corresponding to lowest progression (no Lewy bodies), moderate progression (Limbic type) and advanced progression (Neocortical type) [46].

The OLR method was used to model relationship between the ordinal outcome variables and explanatory predictor variable, namely normalized miRNA counts, using the R package ordinal. The Logit build-in link function was used to determine factors assoicated with Braak, neurofibrillary tangle and plaque density stages. The cummulative link model assumes that thresholds are constant for all values of the explanatory variables. For reported miRNAs, the graphical method for assessing the parallel slopes assumption was used to check ordinal logit requirments. A modified Newton algorithm was used to optimize the likelihood function. The condition number of the Hessian did not indicate a problem with any of the models corresponding to reported miRNAs. Parameter confidence intervals were based on the profile likelihood function, and the estimates in the output are given in units of ordered log odds.

In addition to the usual hypothesis-testing approach, we decided to estimate the effect of a certain variable on the response outcome and its precision. The objective of the model selection analysis is to evaluate whether the effect of the possible predictor is sufficiently important, and as such, determine if it possible to make predictions based on a regression model that includes it as a parameter. Akaike Information Criterion is a particularly useful information theory approach for model selection when a number of variables are believed to have an effect on a process or a pattern.

For the same dataset with the same response variable, the “best” model is the one that minimizes the Kullback-Leibler value, or the information loss when approximating a real process [76]. In order to minimize the expected Kullback-Leibler information, it is necessary to maximize 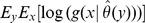 for a collection of admissible models, where *g* is the approximated model in terms of a probability distribution, *y* is the random sample from the density function *f(y)* for the unknown real process *f*, and 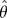 is the maximum likelihood estimate based on the model *g* and data *y* [76]. Approximately unbiased maximum likelihood estimate of 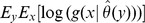 for a large sample corresponds to 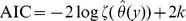, where *k* is the number of estimated parameters included in the model and 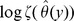 is the log-likelihood of the model given the data, which reflects the overall fit of the model [77]. Essentially, AIC provides an indication of which model would best approximate reality, in terms of minimizing the loss of information, as well as gives a measure of strength of evidence for each model.

For the acquired data, we tested a series of plausible models. The global model, defined as the most complex model considered, was constructed as a set of variables suspected of having an effect on the outcome variable (OLR, uncorrected p-value <0.05, parameter estimate 95% confidence interval did not include zero). Fit of the global model was assessed first. In case of a fit, simpler models, originating from the global model, were compared based on the weight of evidence that model *i* is the best approximation of the true mathematical model given the data and the set of considered candidates [78]. The value of the AIC has no important meaning unless compared to AIC of a series of alternate models. Note that a small Kullback-Leibler information discrepancy in a model corresponds to a small AIC value for the same model. The AIC differences, 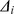, quantify the information loss when one of the fitted models is used instead of the best approximating model. In general, 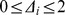 suggests substantial evidence for the model, 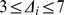 indicates the model has considerably less support, whereas 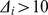 signifies that the model is very unlikely due to essentially no support [78]. We considered predictor variables significant at unadjusted p-value <0.05 and 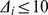.

## Supporting Information

Figure S1 **Consensus clustering of CSF and SER data.** Consensus clustering conjoint with resampling techniques constructs the consensus across multiple runs of a clustering algorithm, determines the number of clusters in the data, and assesses the stability of the generated clusters. Consensus matrices for agglomerative hierarchical clustering upon 1-Pearson correlation distances with 80% item and miRNA resampling was established from log-transformed normalized counts (AD, PD and control combined). Empirical cumulative distribution (CDF) corresponding to the consensus matrices k = {2 (pink), 3 (yellow), 4 (blue), 5 (purple)} was plotted in order to establish stability of the subsequent consensus matrices. Perfect agreement between consensus matrix entries translates into an ideal step function with little shape distortion as k approaches positive infinity. Due to the unimodal nature of the SER consensus matrix histogram, CSF data seems to demonstrate more stable clustering for the first five relevant clusters.
(TIF)

Figure S2 **Distribution of Silhouette scores for the first 15 clusters in CSF and SER data.** Silhouettes quantify how well a data point assigned to a cluster was classified according to both tightness of the clusters and the separation between them. Quality of the cluster assignment, as indicated by the average silhouette score, ranges for 1.0 for unequivocal cluster assignment down to −1.0 for arbitrary assignment. Unsupervised agglomerative hierarchical clustering of CSF and SER data (AD, PD and controls combined) was preformed and average silhouette score was estimated for each cluster. Despite the relatively low silhouette scores, CSF data seems to be more appropriately clustered than SER data, with tighter, more separated clusters.
(TIF)

Table S1 **Demographic information from study subjects.** The samples are color-coded; blue =  subjects with Alzheimer's disease, red =  subjects with Parkinson's disease, yellow =  control subjects. Subject IDs correspond to the data entered into dbGaP, gender (M = 1), APOE status of each subject, disease duration, PMI =  postmortem interval, clinical diagnosis, control, AD =  Alzheimer's disease, PD =  Parkinson's disease, DLB =  Dementia with Lewy Bodies, VAD =  vascular dementia, PSP =  Progressive Supranuclear Palsy, PlaqueF =  frontal lobe, PlaqueT =  temporal lobe, PlaqueP =  parietal lobe, PlaqueH =  hippocampus, PlaqueE =  entorhinal cortex, PlaqueTotal =  sum of scores from all regions, TangleF =  frontal lobe, TangleT =  temporal lobe, TangleP =  parietal lobe, TangleH =  hippocampus, TangleE =  entorhinal cortex, TangleTotal =  sum of scores from all regions, Braak stage, NIA-R =  National Institute of Aging Reagan criteria, LB Stage =  Lewy Body stage, sn_depigmentation (substantia nigra).
(XLS)

Table S2 **Normalized average number of counts per million detected in CSF and Serum from all samples.**
(XLSX)

Table S3 **miRNAs with significant differential expression in serum samples when comparing PD patients and PD with a clinical diagnosis of dementia (PDD).**
(XLS)

Analysis S1 (DOCX)
