# Correction: Profiles of Extracellular miRNA in Cerebrospinal Fluid and Serum from Patients with Alzheimer's and Parkinson's Diseases Correlate with Disease Status and Features of Pathology

**DOI:** 10.1371/journal.pone.0106174

**Published:** 2014-08-13

**Authors:** The PLOS ONE Staff

There is an error in the Supporting Information Analysis S1. Please see the corrected file below.

## Supporting Information

Analysis S1 (DOCX)
