# Supplementary figures and images for: Profiles of Extracellular miRNA in Cerebrospinal Fluid and Serum from Patients with Alzheimer's and Parkinson's Diseases Correlate with Disease Status and Features of Pathology
Source: PLoS One. 2014 May 5;9(5):e94839. doi: 10.1371/journal.pone.0094839 (PMC4010405; doi:10.1371/journal.pone.0094839)

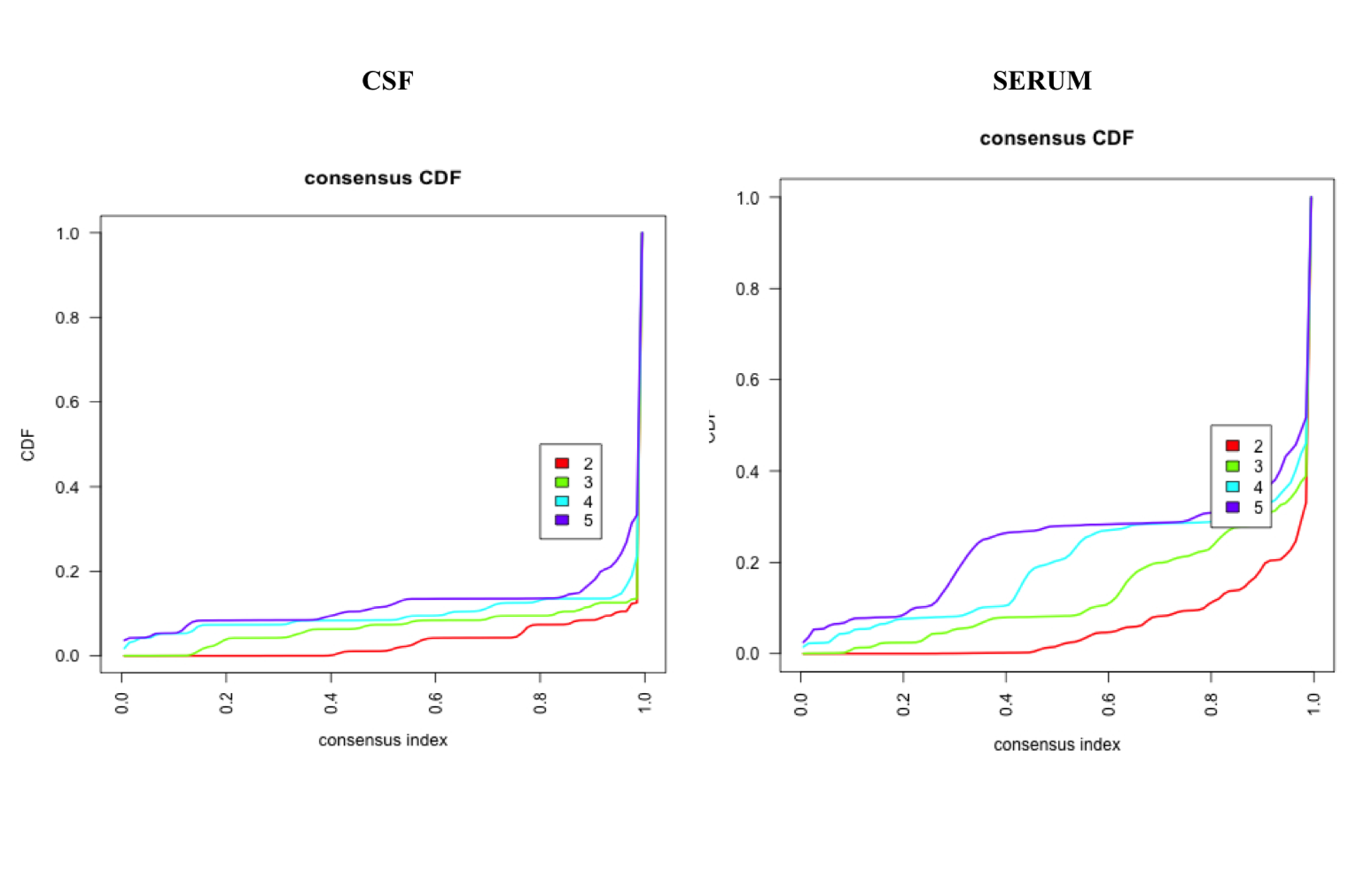

Supplement: Figure S1 — Consensus clustering of CSF and SER data. Consensus clustering conjoint with resampling techniques constructs the consensus across multiple runs of a clustering algorithm, determines the number of clusters in the data, and assesses the stability of the generated clusters. Consensus matrices for agglomerative hierarchical clustering upon 1-Pearson correlation distances with 80% item and miRNA resampling was established from log-transformed normalized counts (AD, PD and control combined). Empirical cumulative distribution (CDF) corresponding to the consensus matrices k = {2 (pink), 3 (yellow), 4 (blue), 5 (purple)} was plotted in order to establish stability of the subsequent consensus matrices. Perfect agreement between consensus matrix entries translates into an ideal step function with little shape distortion as k approaches positive infinity. Due to the unimodal nature of the SER consensus matrix histogram, CSF data seems to demonstrate more stable clustering for the first five relevant clusters. (TIF) [file pone.0094839.s001.tif]

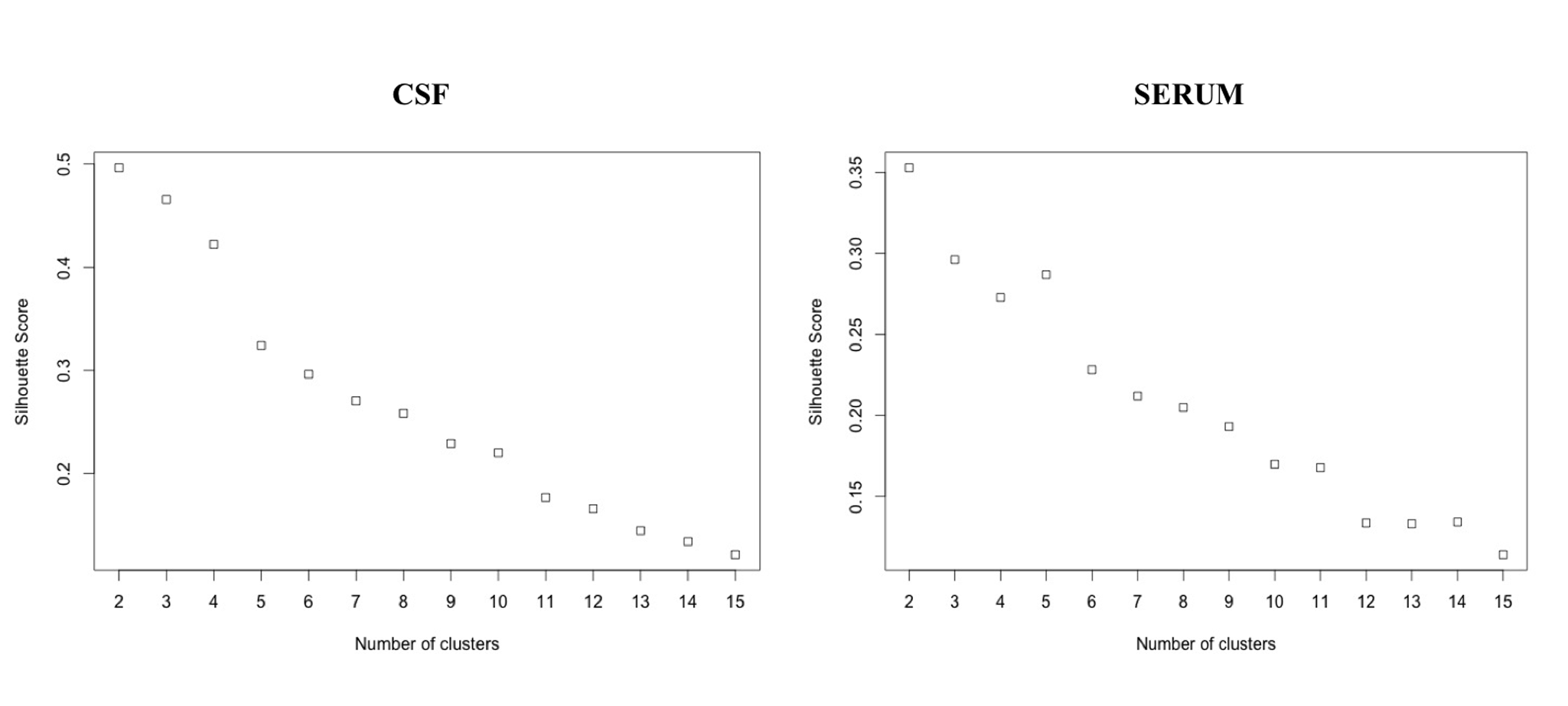

Supplement: Figure S2 — Distribution of Silhouette scores for the first 15 clusters in CSF and SER data. Silhouettes quantify how well a data point assigned to a cluster was classified according to both tightness of the clusters and the separation between them. Quality of the cluster assignment, as indicated by the average silhouette score, ranges for 1.0 for unequivocal cluster assignment down to −1.0 for arbitrary assignment. Unsupervised agglomerative hierarchical clustering of CSF and SER data (AD, PD and controls combined) was preformed and average silhouette score was estimated for each cluster. Despite the relatively low silhouette scores, CSF data seems to be more appropriately clustered than SER data, with tighter, more separated clusters. (TIF) [file pone.0094839.s002.tif]
