# Supplementary material for: Profiles of Extracellular miRNA in Cerebrospinal Fluid and Serum from Patients with Alzheimer's and Parkinson's Diseases Correlate with Disease Status and Features of Pathology
Source: PLoS One. 2014 May 5;9(5):e94839. doi: 10.1371/journal.pone.0094839 (PMC4010405; doi:10.1371/journal.pone.0094839)
Supplement: Analysis S1 — (DOCX) [file pone.0094839.s006.docx]

**SUPPLEMENTARY DATA:**

**Analysis S1. Consensus Clustering and Silhouette Score Analysis**

Consensus clustering is a resampling-based method for assessing stability of the clustering results with respect to sampling variability (Monti et al, 2003). 80% sample and 80% miRNA resampling was used to simulate perturbations of the original datasets, therefore incorporating the relevant dependencies among the observed features. Agglomerative hierarchical clustering upon 1-Pearson correlation distance was then applied to each of the perturbed data sets, and the consensus matrix was assessed. The histogram of a consensus matrix lends itself as a visualization tool for evaluating cluster stability. For a given histogram, we defined and plotted the corresponding empirical cumulative distribution (CDF) for k={2,3,4,5} clusters corresponding to CSF and SER data sets. The underlying motivation for the proposed methodology is that data derived from a more stable biofluid will demonstrate higher robustness to simulated perturbations. The extent to which concentration of a distribution is skewed toward 0 and 1 is a graphical aid indicative of good, stable clustering (Monti et al., 2003). For CSF data, predominance of 0’s and 1’s in the corresponding consensus matrix is reflected in a relatively flat lane across the 0-1 CDF range as $k\to\infty$(**Supplementary Figure S1**). The CDF for SER data on the other hand reflects slightly lesser stability in cluster membership due to a gradual step between 0 and 1, and the clear unimodal nature of its consensus matrix histogram (**Supplementary** **Figure S1**). Note however that the nature of agglomerative hierarchical clustering is such that, even with an absence of detectible multi-cluster structure, the algorithm will still group most of the items according to their distance from each other. Without a multi-cluster signature, most of the items present in the data are assigned to a single cluster (evident as a predominant mode around 1) with a long tail toward 0. In order to address the need for a quantitative measure of how appropriately CSF and SER data have been clustered, we calculated the average silhouette scores for k=[2…15]. Silhouette scores capture suitability of a particular data point to its assigned cluster according to both tightness and separation between clusters (Rousseeuw, 1987). CSF data exemplified higher silhouette scores across the k=[2…15] range, including a higher score for the relevant k=3 number of clusters (**Supplementary** **Figure S2**).

*Consensus Clustering*

Consensus clustering analysis across all conditions contributing one SER and one CSF sample was performed with ConsensusClusterPlus software using log2-transformed normalized count values. Consensus clustering with resampling techniques provides quantitative evidence for determining the number, membership and stability of possible clusters within a dataset (Monti et al., 2003). Resampling technique with 80% sample and miRNA resampling was utilized to simulate perturbations of the original datasets. Agglomerative hierarchical clustering upon 1-Pearson correlation distance was then applied to each of the perturbed subsets, and the consensus among multiple runs, known as the consensus matrix, was assessed. Let *N* be the number of elements in the original dataset *D*. A consensus matrix *M* is an N x N matrix containing the proportion of clustering runs in which two samples are clustered together. If $D^{n}$ is the $n^{th}$perturbed dataset and $C^{n}$is the connectivity matrix representing the result of the clustering algorithm on $D^{n}$, then:

$$C^{n}(i,j)=\left\{_{0\text{, otherwise}}^{1\text{, if samples i and j belong to the same cluster}}\right\}\text{ (1)}$$

Finally, normalized sum of the connectivity matrices of all perturbed subsets of the original dataset constitutes a consensus matrix. If $I^{n}$ is an indicator matrix, then:

$$M(i,j)=\frac{\sum_{1}^{n} c^{n}(i,j)}{\sum_{1}^{n} I^{n}(i,j)}\text{ (2)}$$

Perfect consensus with properly arranged items corresponds to a block-diagonal consensus matrix with non-overlapping blocks of 1’s along the diagonal. Empirical cumulative distribution (CDF) corresponding to consensus matrices k={2,3,4,5} was plotted for both CSF and SER data in order to establish stability of the subsequent consensus matrices. The extend to which CDF is skewed toward 0 and 1 with little shape distortion as k approaches positive infinity is an indication of good, stable clustering (Monti et al., 2003).

*Silhouette Score*

Average silhouette scores for the first k=15 clusters were calculated for datasets consisting of all conditions contributing one CSF and one SER sample. Silhouette scores quantify how well a data point assigned to a cluster was classified according to both tightness of the clusters and the separation between them (Rousseeuw, 1987). The ratio of a point’s dissimilarity to its own cluster to its dissimilarity with its nearest neighboring cluster is captured by the cluster’s silhouette score. Typical range of the score is from negative one, when a point is more similar to members of its neighboring cluster than to other members of its own cluster, to one, for a perfectly classified point (Rousseeuw, 1987). Average of the individual silhouettes constitutes the silhouette score of the entire dataset, and represents the measure of how appropriately the data has been clustered.


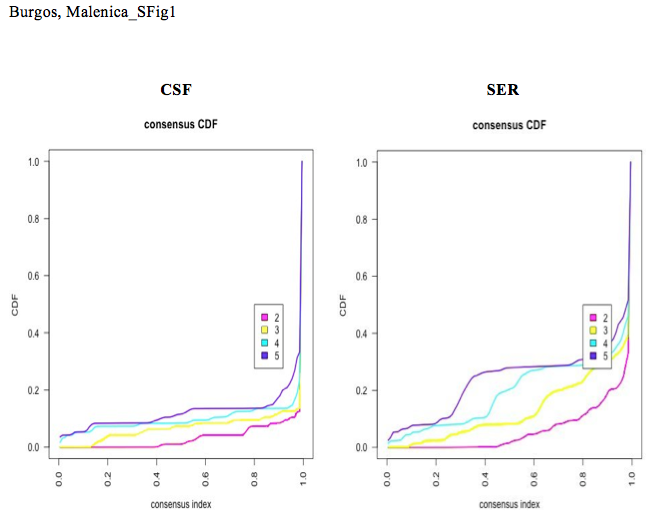


**Supplementary Figure S1. Consensus clustering of CSF and SER data.** Consensus clustering conjoint with resampling techniques constructs the consensus across multiple runs of a clustering algorithm, determines the number of clusters in the data, and assesses the stability of the generated clusters. Consensus matrices for agglomerative hierarchical clustering upon 1-Pearson correlation distances with 80% item and miRNA resampling was established from log-transformed normalized counts (AD, PD and control combined). Empirical cumulative distribution (CDF) corresponding to the consensus matrices k={2,3,4,5} was plotted in order to establish stability of the subsequent consensus matrices. Perfect agreement between consensus matrix entries translates into an ideal step function with little shape distortion as k approaches positive infinity. Due to the unimodal nature of the SER consensus matrix histogram, CSF data seems to demonstrate more stable clustering for the first five relevant clusters.


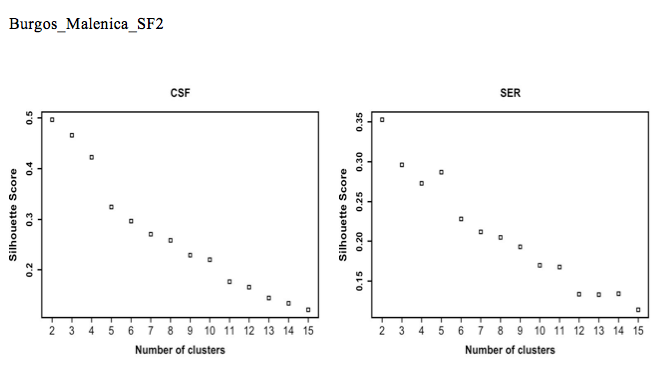


**Supplementary Figure S2. Distribution of Silhouette scores for the first 15 clusters in CSF and SER data.** Silhouettes quantify how well a data point assigned to a cluster was classified according to both tightness of the clusters and the separation between them. Quality of the cluster assignment, as indicated by the average silhouette score, ranges for 1.0 for unequivocal cluster assignment down to -1.0 for arbitrary assignment. Unsupervised agglomerative hierarchical clustering of CSF and SER data (AD, PD and controls combined) was preformed and average silhouette score was estimated for each cluster. Despite the relatively low silhouette scores, CSF data seems to be more appropriately clustered than SER data, with tighter, more separated clusters.
